# Nevirapine and Efavirenz Elicit Different Changes in Lipid Profiles in Antiretroviral- Therapy-Naive Patients Infected with HIV-1

**DOI:** 10.1371/journal.pmed.0010019

**Published:** 2004-10-19

**Authors:** Frank van Leth, Prahpan Phanuphak, Erik Stroes, Brian Gazzard, Pedro Cahn, François Raffi, Robin Wood, Mark Bloch, Christine Katlama, John J. P Kastelein, Mauro Schechter, Robert L Murphy, Andrzej Horban, David B Hall, Joep M. A Lange, Peter Reiss

**Affiliations:** **1**International Antiviral Therapy Evaluation Center, Division of Infectious Diseases, Tropical Medicine, and AIDS, Department of Internal Medicine, Academic Medical Center, University of AmsterdamThe Netherlands; **2**Thai Red Cross AIDS Research CenterBangkokThailand; **3**Department of Vascular Medicine, Academic Medical Center, University of AmsterdamThe Netherlands; **4**Chelsea and Westminster HospitalLondonUnited Kingdom; **5**Fundacion HuespedBuenos AiresArgentina; **6**University HospitalNantesFrance; **7**Sommerset HospitalCapetownSouth Africa; **8**University of CapetownSouth Africa; **9**Holdsworth House General PracticeDarlinghurstAustralia; **10**Hospital Pitie-SalpetriereParisFrance; **11**Hospital Sao Francisco de AssisRio de JaneiroBrazil; **12**Hospital University Clementino Fraga FilohoRio de JaneiroBrazil; **13**Northwestern UniversityChicago, IllinoisUnited States of America; **14**Wojewodzki Szpital ZakaznyWarsawaPoland; **15**Boehringer Ingelheim, RidgefieldConnecticutUnited States of America; St. Vincent's HospitalAustralia

## Abstract

**Background:**

Patients infected with HIV-1 initiating antiretroviral therapy (ART) containing a non-nucleoside reverse transcriptase inhibitor (NNRTI) show presumably fewer atherogenic lipid changes than those initiating most ARTs containing a protease inhibitor. We analysed whether lipid changes differed between the two most commonly used NNRTIs, nevirapine (NVP) and efavirenz (EFV).

**Methods and Findings:**

Prospective analysis of lipids and lipoproteins was performed in patients enrolled in the NVP and EFV treatment groups of the 2NN study who remained on allocated treatment during 48 wk of follow-up. Patients were allocated to NVP (*n =* 417), or EFV (*n =* 289) in combination with stavudine and lamivudine. The primary endpoint was percentage change over 48 wk in high-density lipoprotein cholesterol (HDL-c), total cholesterol (TC), TC:HDL-c ratio, non-HDL-c, low-density lipoprotein cholesterol, and triglycerides. The increase of HDL-c was significantly larger for patients receiving NVP (42.5%) than for patients receiving EFV (33.7%; *p =* 0.036), while the increase in TC was lower (26.9% and 31.1%, respectively; *p =* 0.073), resulting in a decrease of the TC:HDL-c ratio for patients receiving NVP (−4.1%) and an increase for patients receiving EFV (+5.9%; *p <* 0.001). The increase of non-HDL-c was smaller for patients receiving NVP (24.7%) than for patients receiving EFV (33.6%; *p =* 0.007), as were the increases of triglycerides (20.1% and 49.0%, respectively; *p <* 0.001) and low-density lipoprotein cholesterol (35.0% and 40.0%, respectively; *p =* 0.378). These differences remained, or even increased, after adjusting for changes in HIV-1 RNA and CD4+ cell levels, indicating an effect of the drugs on lipids over and above that which may be explained by suppression of HIV-1 infection. The increases in HDL-c were of the same order of magnitude as those seen with the use of the investigational HDL-c-increasing drugs.

**Conclusion:**

NVP-containing ART shows larger increases in HDL-c and decreases in TC:HDL-c ratio than an EFV-containing regimen. Based on these findings, protease-inhibitor-sparing regimens based on non-nucleoside reverse transcriptase inhibitor, particularly those containing NVP, may be expected to result in a reduced risk of coronary heart disease.

## Abstract 2

Comparison of two commonly prescribed non-nucleoside reverse transcriptase inhibitors shows that patients on nevirapine have better blood lipid profiles

## Introduction

Numerous large epidemiological studies have unambiguously demonstrated a strong inverse relationship between the plasma concentration of high-density lipoprotein cholesterol (HDL-c) and the incidence of coronary heart disease (CHD) [1,2]. Recent attempts to develop therapies aimed at increasing HDL-c as innovative CHD-risk-reducing strategies illustrate the potential of HDL-c as a potent anti-atherogenic mediator [3,4,5,6].

Combination antiretroviral therapy (ART) for the treatment of HIV-1 infection has been associated with fat redistribution, insulin resistance, and changes in plasma concentrations of lipids and lipoproteins [7,8,9]. Each of these phenomena is associated with increased CHD risk in the general population. It is not surprising, therefore, that in the setting of HIV-1 infection, increasing exposure to potent combination ART has been demonstrated to be associated with an incremental risk of CHD in a recent prospective study [10]. Interestingly, however, the changes in lipids and lipoproteins differ between patients using an ART regimen containing either a protease inhibitor (PI) or a non-nucleoside reverse transcriptase inhibitor (NNRTI). Whereas many of the PI-based regimens are often associated with increased levels of triglycerides (TGs), total cholesterol (TC), and low-density lipoprotein cholesterol (LDL-c) [8,9,11], NNRTI-based regimens importantly differ from PI-based regimens by being associated with marked increases of HDL-c and lesser increases of LDL-c and TGs [12,13]. Notably, the increases in HDL-c demonstrated with NNRTI-containing ART markedly exceed those that may be induced with any of the currently licensed statins or fibrates [14].

Although as yet no clinical data have been generated to support this, these differences between ART regimens raise the expectation that NNRTI-based regimens, particularly in view of their effects on HDL-c, may favourably modify the CHD risk compared with many of the PI-containing regimens. With respect to the two currently commonly used NNRTIs, nevirapine (NVP) and efavirenz (EFV), no detailed comparative data have been reported concerning their effect on plasma lipids and HDL-c in particular.

We prospectively analysed lipid and lipoprotein changes in a preplanned substudy of the 2NN trial in which ART-naive patients received stavudine (d4T) and lamivudine (3TC) with the randomly assigned addition of NVP, EFV, or both drugs combined.

## Methods

### Participants and Treatment Allocation

The 2NN trial was an open-label study, the main results of which have been published elsewhere [15]. Patients enrolled were 16 y of age or older, ART-naive, and had a plasma HIV-1 RNA concentration (pVL) of at least 5,000 copies/ml. Main exclusion criteria were pregnancy or breastfeeding, abnormal laboratory results at screening, the use of immuno-modulating therapy, or anticipated nonadherence. All patients used d4T (40 mg twice daily [bd] or 30 mg bd when less than 60 kg) and 3TC (150 mg bd). In addition, patients were randomly allocated to NVP at 400 mg once daily (od), NVP at 200 mg bd, EFV at 600 mg od, or NVP and EFV at 400 mg od and 800 mg od, respectively. Patients were included from 65 different study sites in 17 countries in Asia, Australia, North America, South America, South Africa, and Europe. The 2NN study had been approved by the ethics committees of all participating institutions, and all patients had given written informed consent.

The current analyses were preplanned. Only those patients were included who used all components of their allocated treatment for at least 95% of the time during the 48 wk of follow-up (self-reported). Change of d4T and/or 3TC was allowed for reasons of toxicity. Employing such ‘on treatment’ (OT) analysis, allows the best possible assessment of lipid changes that actually result from differences in regimens. Patients in the NVP-od and NVP-bd groups were combined, given that the virologic efficacy of these treatments was comparable and no differences in risk of virologic failure were observed.

### Follow-Up and Assessments

Plasma samples for prospective determination of lipids and lipoproteins were collected at baseline (before start of treatment) and at weeks 2, 4, 8, 12, 24, 36, and 48. Blood was drawn after a mandatory fast of at least 3 h. The samples were analysed in local laboratories according to predefined protocols. These laboratories were selected by the Virtual Central Laboratory (Zeist, The Netherlands), which selected the laboratories, assured the quality of the analyses and data, and standardised all results. Plasma concentrations of HDL-c, TC, and TGs were assessed by standard enzymatic assays. The concentration of LDL-c was calculated using the Friedewald equation, but only when the concentration of TGs was below 4.5 mmol/l [16]. Because the calculation of LDL-c depends on the measured TG concentrations and these TG levels might be biased because of the relatively short mandatory fasting period, we also calculated the non-HDL-c levels. These are considered to be much less influenced by TG levels. The pVL was measured at a central laboratory (LabCorp, Research Triangle Park, North Carolina, United States) using Ultra Sensitive Roche Amplicor 1.5 (Roche Diagnostics, Basel, Switzerland) with a lower limit of quantification of 50 copies/ml.

### Outcome Measurements

The primary study outcome was the mean percentage change of HDL-c, TC, TC:HDL-c ratio, non-HDL-c, LDL-c, and TGs between start of allocated treatment and week 48. For each patient at each specific study week we calculated this estimate as concentration at week *X* minus concentration at baseline divided by concentration at baseline, times 100. Study-week-specific estimates were used for the subsequent analyses.

Factors assessed for a possible association with the primary outcome were sex, study region (Asia/Australia, South Africa, South America, Europe/North America), body mass index (BMI) (continuous), increase between start of therapy and week 48 in CD4+ cells (<100, 100–250, or >250 cells/mm^3^) or decrease in pVL (<2.5, 2.5–3.5, or >3.5 log_10_), and virologic failure during follow-up. Virologic failure was defined as (1) never having obtained a pVL of less than 50 copies/ml or (2) a rebound to two consecutive pVLs of ≥50 copies/ml. A single pVL of ≥50 copies/ml at week 48 was also considered a virologic failure.

### Statistical Analyses

The analyses included the NVP and EFV treatment groups only. This choice was made since the results of the main 2NN study clearly showed that the simultaneous use of NVP and EFV shouldn't be recommended in clinical practice in view of increased toxicity of this combination in the absence of increased virologic efficacy.

The mean percentage changes in lipid concentrations were modelled using a mixed model incorporating repeated measurements. This model handles missing data adequately by estimating the outcome of a specific variable based on the available data given the specified covariate structure. The variables (fixed effects) in the model were tested for significance using the Type III F-statistic. The estimates of a specific level of the fixed effect were modelled using the ‘least squared means' approach. Differences in these estimates between different levels of the variable were tested for significance using the t-statistic.

Since the analyses might be biased because of the OT approach or the modelling of data, we performed two sensitivity analyses. The first was an analysis using the same modelling strategy but for an intention-to-treat population including all patients who started their randomised treatment. The second was an analysis using only available data for the OT population, without modelling of data points.

Independent risk factors were assessed by multivariable regression analyses. The multivariable analysis included the variable ‘treatment group' and all predefined variables. Interaction between treatment group and a specific variable was assumed at a *p*-value less than 0.15. A two-sided *p*-value less than 0.05 was considered statistically significant in the final analyses. The SAS statistical package was used for analyses (version 8.02, SAS Institute, Cary, North Carolina, United States).

## Results

### Disposition of Patients

Of the 1,216 patients included in the 2NN study, 607 were allocated to the NVP treatment group and 400 to the EFV treatment group. Of these, 42 (6.9%) patients in the NVP group and 25 (6.3%) patients in the EFV group did not start their treatment or were considered a ‘study entry violator’ by a (blinded-to-treatment) independent endpoint committee. These patients were excluded from the analyses. From the remaining patients (565 using NVP and 375 using EFV), only those who remained on their assigned treatment during the follow-up were included in the analyses. This resulted in a final sample size of 417 (68.7%) patients in the NVP group and 289 (72.3%) in the EFV group.

All of the included patients had at least one measurement of each lipid parameter and could therefore be used in the statistical models. The baseline characteristics of the subset of patients included in the current analyses are summarised in Table 1. These baseline characteristics were comparable with those of all patients enrolled in the main 2NN study.

**Table 1:**
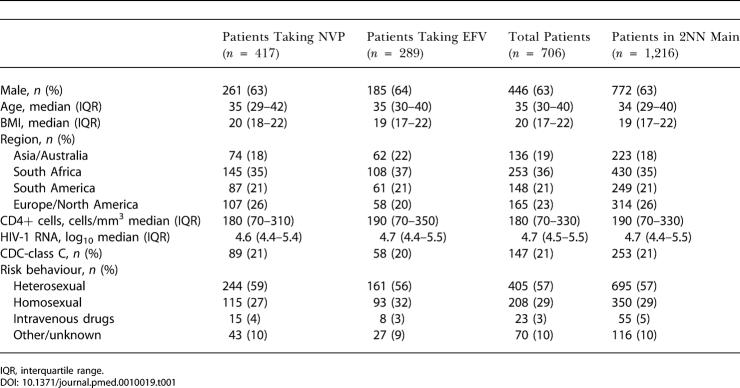
Baseline Characteristics of Patients Included in the 2NN Lipid Substudy and the 2NN Main Study

IQR, interquartile range

In the NVP group, 148 of the 565 eligible patients (26.2%) were not included in the OT analyses. Of these 96 (65%) were nonadherent (including patients lost to follow-up while on randomised treatment), 30 (20%) changed their NNRTI to EFV, and 22 (15%) changed their regimen by adding a PI. In the EFV group, 76 of the 375 eligible patients (20.3%) were not included in the OT analyses. Of these 54 (71%) were nonadherent, 17 (22%) changed their NNRTI to NVP, 3 (4%) added a PI, and 2 (3%) added a third nucleoside reverse transcriptase inhibitor.

### Changes in Lipids and Lipoproteins

All changes within the treatment groups in lipid and lipoprotein concentration, as well as in TC:HDL-c ratio were statistically significant.

The increase of HDL-c was 8.9% (95% confidence interval [CI], 0.6–17.1) larger in the NVP treatment group (42.5%) than in the EFV treatment group (33.7%). This was statistically significant (*p =* 0.036) (Table 2). In contrast, the increase in TC was smaller in the NVP group (26.9%) than in the EFV group (31.1%), but this difference (−4.2%; 95% CI, −8.7 to 0.4) was not statistically significant (*p =* 0.073). These changes resulted in a decrease of the TC:HDL-c ratio in the NVP group (−4.1%) compared to an increase in the EFV group (+5.9%; *p <* 0.001), and a significantly smaller increase of non-HDL-c in the NVP group (difference, −8.9%; 95% CI, −15.4 to −2.5; *p =* 0.007).

**Table 2:**
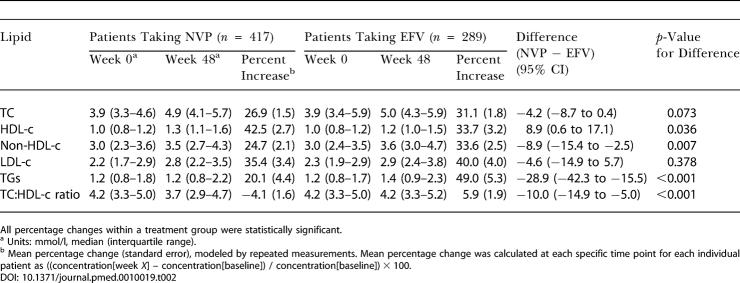
Lipid Concentrations at Baseline and Week 48 and Mean Percentage Change

All percentage changes within a treatment group were statistically significant

^a^ Units: mmol/l, median (interquartile range)

^b^ Mean percentage change (standard error), modeled by repeated measurements. Mean percentage change was calculated at each specific time point for each individual patient as ((concentration[week *X*] – concentration[baseline]) / concentration[baseline]) × 100

The increase of TGs was 28.9% (95% CI, −42.3 to −5.0) smaller in the NVP group (20.1%) than in the EFV group (49.0%; *p <* 0.001). The difference in LDL-c increase was not statistically significant (35.4% for NVP group; 40.0% for EFV group; *p =* 0.378).

In the first sensitivity analysis (intention-to-treat population), the increases of HDL-c were slightly lower (41.2% for NVP group; 32.4% for EFV group), just as for TC (26.1% for NVP group, 30.4% for EFV group) and non-HDL-c (24.3% for NVP group, 33.1% for EFV group). The TC:HDL-c ratio showed a smaller decrease for patients taking NVP (−2.6%) but a larger increase for patients taking EFV (+7.2%). The increase in TGs was larger for both patients taking NVP (24.3%) and patients taking EFV (49.3%). The LDL-c increase was somewhat smaller for patients taking NVP (33.1%) but larger for patients taking EFV (47.3%).

The difference between patients taking NVP and those taking EFV for HDL-c (8.8%; 95% CI, 1.3−16.3) remained statistically significant, just as the difference in the TC:HDL-c ratio (−9.8%; 95% CI, −14.7 to −4.9), non-HDL-c (−8.8%; 95% CI, −14.6 to −3.0), and TGs (−24.9%; 95% CI, −37.2 to –12.6). Additionally, the difference between NVP and EFV treatment groups became statistically significant for TC (−4.2%; 95% CI, −8.5 to 0.0) and LDL-c (−14.2%; 95% CI, −28.4 to 0.0) compared to the original OT analysis. The second sensitivity analysis (using only available data for the OT population) also showed comparable estimates (data not shown).

The increase in HDL-c for patients who started their ART when their HDL-c levels were, according to the National Cholesterol Education Program (NCEP) guidelines, low (<1.03 mmol/l), normal (1.03–1.55 mmol/l), or high (>1.55 mmol/l) is reported in Table 3. In both treatment groups, the majority of patients had a low HDL-c at the start of therapy. These patients showed the largest increase in HDL-c over 48 wk. Even patients with a normal baseline HDL-c level showed statistically significant, marked increases of HDL-c. The effect of baseline HDL-c level on percentage increase was comparable in both treatment groups (interaction, *p =* 0.409).

**Table 3:**
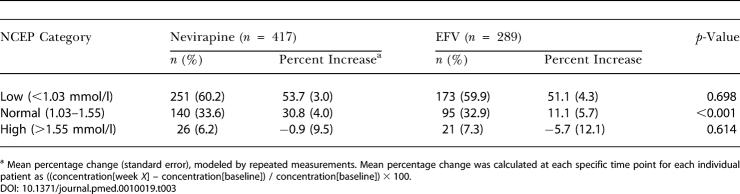
Increase in HDL-c Stratified by Baseline HDL-c (NCEP Categories)

^a^ Mean percentage change (standard error), modeled by repeated measurements. Mean percentage change was calculated at each specific time point for each individual patient as ((concentration[week *X*] – concentration[baseline]) / concentration[baseline]) × 100

### Multivariable Analysis

Factors independently associated with changes in the lipid concentrations were analysed by a multivariable regression analysis (Table 4).

**Table 4:**
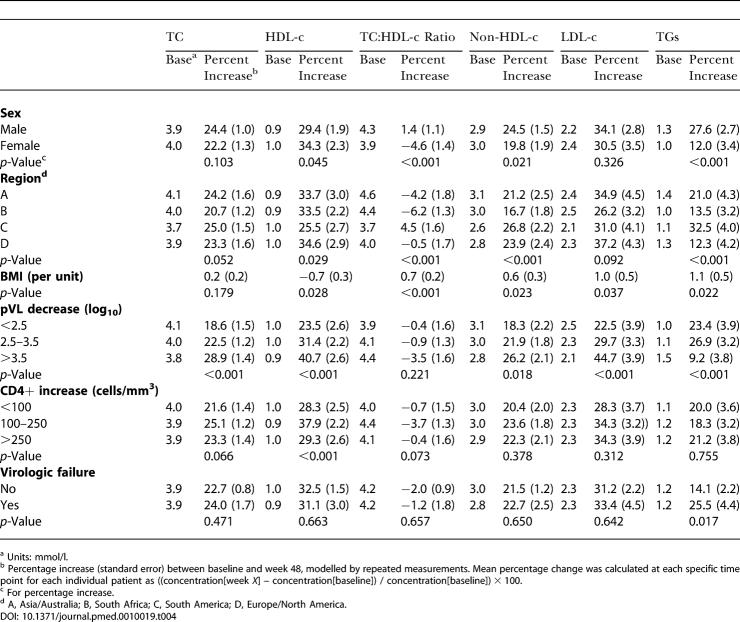
Factors Associated with Percentage Change in Lipid Parameters (Multivariable Analyses)

^a^ Units: mmol/l

^b^ Percentage increase (standard error) between baseline and week 48, modelled by repeated measurements. Mean percentage change was calculated at each specific time point for each individual patient as ((concentration[week *X*] – concentration[baseline]) / concentration[baseline]) × 100

^c^ For percentage increase

^d^ A, Asia/Australia; B, South Africa; C, South America; D, Europe/North America

Men had a significantly smaller increase of HDL-c, compared to women, but a larger increase of TC. This resulted in an increased TC:HDL-c ratio for men and a decreased ratio for women, while the increase of non-HDL-c was significantly larger in men.

The changes in lipid concentrations varied markedly by region. Patients from Asia/Australia and South Africa had the largest decrease in the TC:HDL-c ratio because of an increase of HDL-c that outweighed the increase of TC. Patients from South America, compared to those from other regions, had a significantly smaller HDL-c increase with a comparable TC increase, resulting in an increased TC:HDL-c ratio. Although patients from Europe showed the largest change in HDL-c, the increases in TC and TC:HDL-c ratio were intermediate. A striking finding is the much larger increase of TGs in patients from South America compared to those in patients from other regions, which to a lesser extent was also seen for non-HDL-c.

For all lipid concentrations, except TGs, there was a clear pattern of larger increases of lipid levels with larger decreases of pVL over 48 wk. This was also seen when the pVL increase over 48 wk was analysed as a continuous variable. For each log_10_ larger decrease in pVL there was a 4.6% increase in TC (*p <* 0.001), a 7.8% increase in HDL-c (*p <* 0.001), a 10.2% increase in LDL-c (*p <* 0.001), and a 3.6% increase in non-HDL-c (*p =* 0.002), while the TC:HDL-c ratio declined with 1.6% (*p =* 0.051). In this analysis, there was also a clear association between pVL decline and change in TGs (6.3% decline per log_10_; *p =* 0.002).

In general, a smaller CD4+-cell increase was associated with a smaller increase in lipid concentration, while increases of more than 250 cells/mm^3^ did not show markedly different effects compared to increases of 100–250 cells/mm^3^. When analysed as a continuous variable, there was no statistically significant association between CD4+-cell increase and change in any of the lipid parameters.

BMI was independently associated with increases in all lipid parameters, except TC. Although the increases per unit increase in BMI were statistically significant, the magnitude of increases was rather low.

All these factors exhibited a similar effect in both the NVP and the EFV treatment group (no significant interactions). There were, however two exceptions. For changes in TGs, the effect of sex and pVL decrease differed between the treatment groups (interaction, *p =* 0.005 and *p =* 0.075, respectively). Men had a significant increase of TGs in both the NVP group (14.1%) and the EFV group (43.3%); women using NVP had no significant TG increase (6.5%), while those using EFV had (15.9%). The effect of pVL decrease on TG increase was quite different for patients taking NVP versus those taking EFV. In the NVP group, the increase in TG concentration was 17.2% for a pVL decrease less than 2.5 log_10_, 16.6% for a decrease between 2.5 and 3.5 log_10_, and −6.1% (denoting a decrease) for a pVL decrease more than 3.5 log_10_. In the EFV group, these estimates were 27.4%, 37.2%, and 26.0%, respectively.

Adjusting for the variables included in the multivariable model, the difference between patients taking NVP and those taking EFV in HDL-c increase (9.8%; 95% CI, 3.4−16.3) and decrease of the TC:HDL-c ratio (−11%; 95% CI, −15.1 to −6.8) remained statistically significant.

Also, the difference in non-HDL-c increase (−9.5%; 95% CI, −14.6 to −4.4) and TG increase (−27.2%; 95% CI, −38.0 to −16.4) remained statistically significant.

The difference in TC increase (−4.4%; 95% CI, −8.0 to −0.8) became statistically significant. The difference between NVP and EFV groups for the increase in LDL-c remained statistically nonsignificant (−6.1%; 95% CI, −14.7 to 2.6).

The adjusted increase of HDL-c was 42.3% and 32.4% for patients taking NVP and EFV, respectively (*p =* 0.003). The adjusted change in TC:HDL-c ratio was −4.3% for patients taking NVP and +6.6% for patients taking EFV (*p* < 0.001). These values were 26.6% and 31.0% for TC (*p =* 0.020), 24.4% and 33.9% for non-HDL-c (*p <* 0.001), 17.9% and 45.1% for TG (*p* < 0.001), and 35.5% and 41.5% for LDL-c (*p =* 0.168). These estimates were very similar in the two sensitivity analyses (data not shown).

**Figure 1:**
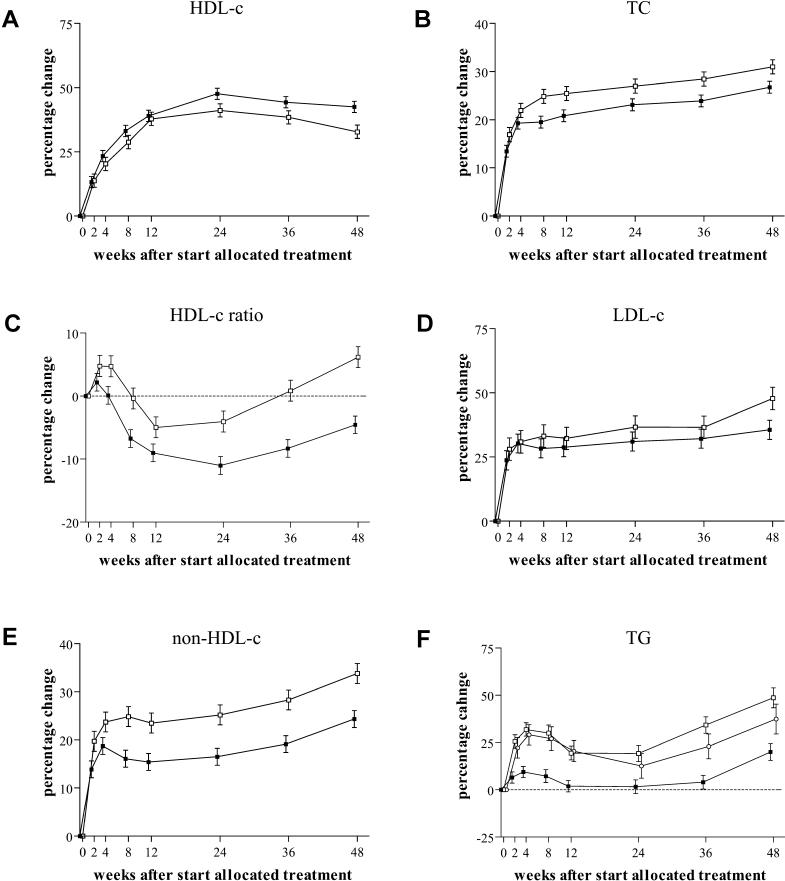
Change in Plasma Concentrations of Lipids and Lipoproteins Adjusted for sex, region, pVL decrease, and CD4+-cell increase.

The proportional changes of the different plasma lipid concentrations over 48 wk are graphically depicted in Figure 1.

## Discussion

Initiation of an ART regimen containing NVP or EFV is accompanied by a significant increase of HDL-c, with concomitant increases of TC, non-HDL-c, TGs, and LDL-c. The proportional increase of HDL-c was significantly larger in the NVP treatment group compared to the EFV treatment group, while the proportional increase of TC, non-HDL-c, and TGs was significantly smaller. In the NVP group, the TC:HDL-c ratio decreased, compared to an increase in the EFV group. These observations are different from what is seen with most PI-based ART regimens, in which higher concentrations of TC, LDL-c, and TGs are reported but without the concurrent higher levels of HDL-c [17,18].

In contrast to a small randomised study (*n =* 67) that did not show significant differences between NVP and EFV [19], the present study demonstrates a more favourable lipid profile for treatment including NVP than for treatment including EFV in ART-naive patients.

### HDL-c Increase and NNRTI

Increases of HDL-c with the use of NVP or EFV have been described in studies for patients switching from a PI-based regimen to a NNRTI-based regimen [20,21]. Data for ART-naive patients starting therapy with an NNRTI-based regimen are scarce. Van der Valk et al. reported an increase of HDL-c of 0.44 mmol/l for patients initiating treatment with didanosine, d4T, and NVP in the Atlantic trial [12]. Tashima et al. reported an increase of HDL-c of 0.21 mmol/l in patients treated with EFV and either zidovudine plus 3TC, or indinavir [13], while Negredo et al. showed an increased HDL-c concentration in therapy-naive patients starting a regimen of didanosine, d4T, and EFV (0.34 mmol/l) [22].

The present study showed a clear effect of baseline HDL-c on the proportional increase. The largest increases were seen for patients who had an increased CHD risk based on their low HDL-c level (<1.03 mmol/l) according to NCEP guidelines. But also patients with a normal HDL-c level, who are not at an increased CHD risk, showed marked increases in HDL-c. This baseline effect can likewise be distilled from the other studies, where those with the lowest baseline value (0.93 mmol/l; Atlantic study [12]) showed the largest HCL-c increase, while the smallest increase was seen in the study with the highest baseline value (1.23 mmol/l; Tashima study [13]). The study by Negredo et al. [21], which included patients with similar baseline HDL-c levels as in the present study, showed an increase of HDL-c comparable to that in the present study (0.34 and 0.36 mmol/l, respectively). The much more modest HDL-c-increasing effect of statins likewise shows such a correlation with baseline level in patients without HIV-1.

One may postulate that the HDL-c increase merely reflects an adequate suppression of HIV-1 infection (‘return towards normal’). In support, a larger decrease in pVL was associated with a larger increase of HDL-c in the present study. However, the magnitude of the HDL-c increase was only slightly different for patients experiencing virologic failure during these 48 wk (29.5%) and those with complete suppression (34.0%; *p =* 0.161), and the increases of HDL-c remained statistically significant even after adjustment for pVL decrease.

Riddler et al. compared changes in lipid concentrations before seroconversion for HIV-1, initiation of ART, and during ART in patients using different ART regimens, which all but one included a PI [23]. The period between seroconversion and start of ART was characterised by decreases in TC, LDL-c, and HDL-c.

Between initiation of ART and the first follow-up visit (mean, 1.3 years), the concentrations of TC and LDL-c increased again to levels that did not differ significantly from before seroconversion. Such a ‘return to normal’ as a result of ART was not seen for HDL-c. The reported increases by Riddler et al. in TC (0.88 mmol/l) and LDL-c (0.41 mmol/l) were of a comparable magnitude to that found for patients taking NVP in this present study (0.97 and 0.55 mmol/l, respectively). However, the HDL-c increase was more than ten times smaller (0.03 mmol/l) in the Riddler et al. study than the 0.36 mmol/l observed in patients taking NVP in the present study, while the mean HDL-c values at which ART was started were comparable in the two studies (1.04 and 1.0 mmol/l, respectively). This indicates that although at least part of the change in TC and LDL-c may reflect a ‘return towards normal’, the magnitude of the HDL-c increase observed in our study must have occurred through additional mechanisms. Since we have no information on the antiretroviral efficacy of the regimens used in the Riddler et al. study, we have to consider that the reported differences between the Riddler et al. study and the present study might be partly due to differences in HIV-1 suppression. However, the type of PI-based regimens used in the Riddler et al. study and the long-term adequate adherence by the patients make large differences in antiretroviral efficacy unlikely.

We are currently conducting studies to unravel whether NVP possibly stimulates synthesis of the most important apolipoprotein of HDL-c, apoAI, or alternatively, for instance, decreases the clearance of HDL-c particles.

Several studies have convincingly shown that an HDL-c increase is associated with a significant decrease in CHD mortality independent of changes in LDL-c [1,2]. Overall, extrapolation of these studies indicates that a 0.025-mmol/l increase in HDL-c is expected to be associated with a 2%–3% reduction in CHD risk, while an increase of 1.0 mmol/l in LDL-c will increase the CHD risk by 25%. The mean absolute increases in HDL-c and LDL-c were 0.36 and 0.54 mmol/l, respectively, for patients taking NVP, and 0.24 and 0.65, respectively, for patients taking EFV. It can therefore be estimated that, taking the observed effects on both HDL-c and LDL-c into account, the reduction in CHD risk would be 15% for patients taking NVP and 3% for patients taking EFV compared to ART regimens that do not include NNRTIs. Although the differences in absolute concentrations of HDL-c and LDL-c may seem modest when comparing the NVP and EFV treatment groups, the combined effect of these changes on CHD risk seems marked. It should be emphasised that these are theoretical estimates, which do not take into account that increases in TGs would be expected to have an opposite effect on CHD risk. The increase in this last parameter is, however, smaller for patients taking NVP than for patients taking EFV. Furthermore, we do not have information on the presence of conventional risk factors for CHD. The actual effect of the lipid changes associated with particular ART regimens on CHD can only be substantiated by clinical endpoint studies.

### Changes in TGs, TC, and LDL-c

The data indicate that EFV might have a more detrimental effect on TG levels than NVP. That EFV indeed can be associated with an increase in TGs was shown in two studies, in which sporadical hypertriglyceridaemia was reported in patients starting an ART regimen with EFV but without d4T [24,25]. A difference between NVP and EFV treatment with respect to the TG effect is also in line with a study by Negredo et al. [20]. In this study patients were randomised to either continue their successful PI-based regimen or to change to an NVP-based or an EFV-based regimen. Only patients switching to the NVP regimen showed a significant decrease in TG levels.

The proportional increase in TG levels with both NVP and EFV treatment seems large, but the median absolute TG level at week 48 was still low in both treatment groups (1.2 mmol/l and 1.4 mmol/l, respectively). In the NCEP guidelines, a TG concentration below 1.69 mmol/l is still considered normal [26]. The increase in TG level is therefore probably not clinically meaningful.

The differences between patients taking NVP and those taking EFV in changes in TC as well as TGs are unlikely to be explained by the concurrent use of d4T. In both treatment groups, the percentage of patients who used d4T as part of their regimen throughout follow-up was high (96% for the NVP treatment group and 98% for the EFV treatment group). This high rate of d4T use might, however, be responsible for the impression that the increases in TC and TGs seem to continue or even accelerate towards the end of the study period, as opposed to a somewhat declining effect of treatment on HDL-c after 24 wk. A possible explanation for this may be the gradual, progressive worsening of fat redistribution or lipodystrophy that one would expect to occur in this continuously d4T-exposed patient population. Both the incidence and severity of lipodystrophy are particularly increased with d4T-containing ART regimens, and lipodystrophy has been reported to be associated with increased TC and TG levels [27,28,29,30,31,32]. It is therefore conceivable that the lipid changes in the second part of the study represent a combined effect of the NNRTI used and a superimposed effect resulting from gradually worsening fat redistribution.

Due to the relatively short mandatory fasting period (3 h), the measured TG concentration might be biased, possibly even more so given that HIV-1 infection may be associated with reduced TG clearance following food intake [33]. As a consequence, the estimates of calculated LDL-c might be biased. TG levels influence changes in non-HDL-c less. The fact that in the present study the increases of non-HDL-c, LDL-c, and TGs are all smaller for patients taking NVP than for patients taking EFV suggests that the LDL-c and TG results are valid despite the potentially short mandatory fasting periods.

### ART and CHD

The relationship between ART and CHD has been the subject of several studies, based on either clinical or validated surrogate endpoints (like arterial intima-media thickness [34] or endothelial wall function [35]).

Studies examining intima-media thickness in patients with HIV-1 treated with ART, or more specifically with PI-based ART, remain inconclusive as to whether ART use induces accelerated intima-media thickening [36,37,38,39]. PI use may have a detrimental effect on endothelial function in vivo, or accelerate foam cell formation in vitro [40,41].

In a retrospective clinical endpoint study including almost 37,000 patients, Bozzette et al. reported no relation between ART use and hospital admission for cardiovascular events [42], a finding confirmed in the ‘Kaiser Permanente’ cohort [43]. However, the large prospective ‘data collection on adverse events of anti-HIV drugs' (D:A:D) study, specifically designed to identify the extent to which ART may be associated with increased CHD risk, did show that every additional year of ART use was associated with a 26% increased risk of myocardial infarction [10]. The latter resembles the results from other retrospective and prospective studies [44,45,46]. The relatively high prevalence of known CHD risk factors in patients with HIV-1, especially smoking [47,48], complicates interpretation of the relation between ART use and CHD. None of these studies allows definitive conclusions to be made about the potentially different degree of risk associated with particular ART regimens.

### Limitations and Possible Biases

The selection of patients remaining on their allocated treatment for the full 48 wk might have introduced sampling bias with inflated treatment effects. The reported estimates from this OT analysis were very similar to the estimates from the intention-to-treat analysis. This is caused by the fact that only a few patients who were not included in the OT analyses changed their drug regimen by adding a PI that could potentially influence the lipid estimates. The majority remained on their randomised treatment but were insufficiently adherent (or lost to follow-up on their original regimen) to meet the criteria for being eligible for the OT analysis. Patients replacing their assigned NNRTI by the NNRTI from the other treatment group of the study would have little effect on the lipid estimates, since both NNRTIs show changes in lipid concentrations, which go in the same direction. Another possible reason for the similarity between these two analyses could be the relatively late timing of treatment changes (mean of 75 d for patients taking NVP and 95 d for patients taking EFV). The fact that the second sensitivity analysis also showed comparable results indicates that modelling of data did not affect the lipid estimates.

A limitation of the present study is the lack of data on conventional CHD risk factors like smoking. The 2NN being a randomised study, it may be expected that these and other confounding variables are equally distributed over the treatment groups. Possible residual confounding, however, cannot be excluded, and the results should therefore be interpreted cautiously.

### Conclusion

While awaiting the results of future studies, the less atherogenic lipid profile of patients taking NVP in comparison to those of patients taking EFV may be among the various factors to consider when selecting the most appropriate initial ART regimen, particularly for those patients with HIV-1 with a significant a priori CHD risk. Including such a consideration seems warranted, since treatment of the ART-induced lipid changes with currently licensed lipid-lowering agents is not without problems. Most of the available statins except pravastatin and fluvastatin, are metabolised through cytochrome isoenzyme CYP3A4, just as the PIs and NNRTIs are, providing concern for potential drug–drug interactions. Furthermore, statin therapy in patients using a PI-based regimen is in general not able to reduce the lipid concentrations to normal levels [49,50]. Studies on the effectiveness of gemfibrozil in patients on a PI-based regimen show conflicting results [51,52,53]. Finally, the introduction of yet another type of medication in a patient population that often needs to use not only ART but also a considerable amount of concomitant medication might jeopardise treatment adherence, which is of crucial importance for the sustained success of ART treatment.

Use of the novel PI atazanavir may also be considered an attractive option in patients at high risk of CHD, given that it is associated with markedly smaller increases of TC, LDL-c, and TGs compared to previously available PI-based regimens [54,55], but it lacks the concurrently large increase in HDL-c seen with NNRTI-based regimens.

The reported increase of HDL-c concentration with NNRTI use is far greater than that seen with conventional lipid-lowering drugs and is of a similar magnitude as the HDL-c increases reported with the most powerful HDL-c-increasing drugs that are currently in clinical development [6,56]. Asztalos et al. reported the effect on HDL-c for five major statins in patients with CHD [57]. They concluded that the HDL-c increase was between 4% (simvastatin) and 11% (pravastatin and lovastatin). Treatment with fluvastatin or lovastatin proved to be most effective in patients with a low baseline HDL-c level [56,58]. Clinical trials assessing the effects of fibrates reported an HDL-c increase of 6% and 11% for gemfibrozil [2,59], and 18% for bezafibrate [60].

Unravelling the mechanism or mechanisms by which NVP and EFV raise HDL-c could contribute to the development of novel interventions aimed at increasing HDL-c and thereby ultimately to reducing CHD risk in the population at large.

**Patient Summary**

**Why Did the Researchers Do the Study?**

Drugs used to treat HIV (antiretroviral drugs) help patients to live longer, but they can also have some serious side effects. For example, the longer people take them, the higher the risk they'll get heart disease. Why? Part of the reason is that many—though not all—antiretroviral drugs cause changes in cholesterol levels in the bloodstream (an increase in the amount of “bad” cholesterol and a reduction in the amount of “good” cholesterol).

Two of the most commonly prescribed antiretroviral drugs are nevirapine and efavirenz. Previous smaller studies showed that treatment with either of the drugs could increase the amount of “good” cholesterol. The researchers now wanted to directly compare these drugs to find out what effect they had on patients' cholesterol levels in a much larger group of patients.

**What Did the Researchers Do?**

The scientists studied adults with HIV who had never previously taken antiretroviral drugs. All of the patients then took “triple therapy”—a combination of three antiretroviral drugs. Some of the patients took nevirapine as part of their triple therapy, whereas some took efavirenz. The researchers took blood samples regularly for almost a year and measured patients' cholesterol levels.

**What Did the Researchers Find?**

They confirmed that both nevirapine and efavirenz indeed have a beneficial effect on patients' cholesterol levels. They both increase the amount of “good” cholesterol in the bloodstream. The increase was higher with nevirapine than with efavirenz.

**What Does This Study Mean for Patients?**

If your treatment includes nevirapine or efavirenz (particularly nevirapine), this can raise your level of “good” cholesterol.

The results of the study may be especially important if you are already at risk for heart disease. In other words, if you have risk factors for heart disease—high blood pressure, diabetes, heart disease running in your family, being a smoker—it may be beneficial for your HIV medications to include nevirapine. If you smoke, you can lower your risk of heart disease by quitting. If you have high blood pressure or diabetes, treating these conditions can also lower your risk of heart disease.

**What Are the Problems with the Study?**

Although the researchers showed that nevirapine and efavirenz can have a beneficial effect on cholesterol levels, they haven't actually shown that this reduces patients' risk of getting heart disease.

The study was funded by the company that produces nevirapine. In theory this could have affected the results (research has shown that company-sponsored studies are more likely to produce results favorable to the company than studies without sponsorship). The study, however, was carried out by a network of independent investigators who state that the company had no influence on the reporting of the results.

**Where Can I Get More Information?**

You can get more information on HIV and its treatment from the Terrence Higgins Trust (www.tht.org.uk), AIDS.ORG (www.aids.org), and The Body (www.thebody.com).

## Abbreviations

3TC: lamivudine
ART: antiretroviral therapy
bd: twice daily
BMI: body mass index
CHD: coronary heart disease
CI: confidence interval
d4T: stavudine
EFV: efavirenz
HDL-c: high-density lipoprotein cholesterol
LDL-c: low-density lipoprotein cholesterol
NCEP: National Cholesterol Education Program
NNRTI: non-nucleoside reverse transcriptase inhibitor
NVP: nevirapine
od: once daily
OT: on treatment
PI: protease inhibitor
pVL: plasma HIV-1 RNA concentration
TC: total cholesterol
TG: triglyceride
