# Correction: Nevirapine and Efavirenz Elicit Different Changes in Lipid Profiles in Antiretroviral-Therapy-Naive Patients Infected with HIV-1

**DOI:** 10.1371/journal.pmed.0010073

**Published:** 2004-12-28

Published October 19, 2004

**In *PLoS Medicine,* volume 1, issue 1:**

Nevirapine and Efavirenz Elicit Different Changes in Lipid Profiles in Antiretroviral-Therapy-Naive Patients Infected with HIV-1

**Frank van Leth, Prahpan Phanuphak, Erik Stroes, Brian Gazzard, Pedro Cahn, et al.**

DOI: 10.1371/journal.pmed.0010019

The following information was missing from the legend for Figure 1: closed squares, NVP; open squares, EFV; error bars denote standard errors. Oliver Flint at Bristol-Myers Squibb alerted PLoS Medicine to this omission.

**Figure 1:**
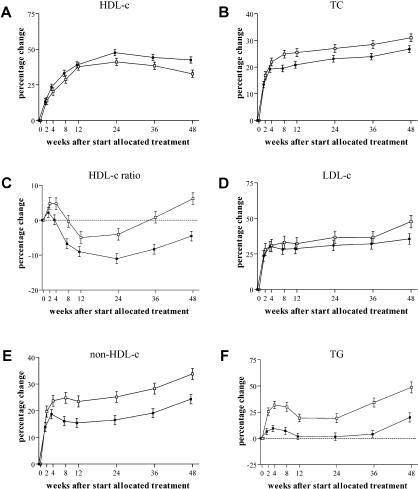
Change in Plasma Concentrations of Lipids and Lipoproteins Adjusted for sex, region, pVL decrease, and CD4^+^-cell increase. Closed squares, NVP; open squares, EFV; error bars denote standard errors.

There was also a line with open circles in Figure 1F that should not have been included.

